# 

**Published:** 1879-06

**Authors:** 


### Books Received.

Hearing and How to Keep It. By Charles H. Bennett, M. D. Philadelphia: Lindsay & Blakiston.

First of a series of small volumes on subjects pertaining to sanitary and the preservation of health, by American Authors.

Cyclopædia of the Practice of Medicine. Edited by H. Von Ziemssen, Vol. VIII. Diseases of the Chylopœtic System, with chapters relating to diseases of the Bladder and Urethra and functional affections of the male genital organs. New York: William Wood & Company, 27 Great Jones St. Buffalo: J. H. Mattison.

Demonstrations of Anatomy, being a guide to the knowledge of the human body by dissection. By George Viner Ellis, Prof. of Anatomy in University College, London. Illustrated by two hundred and forty-nine engravings on Wood. Philadelphia: Henry C. Lea, 1879.

A Guide to Therapeutics and Materia Medica. By Robert Farquharson, M. D., Edin., F. R. C. T. London. Enlarged and adapted to the U. S. Pharmacopœia, by Frank Woodbury, M. D. Philadelphia: Henry C. Lea, 1879. Buffalo: T. H. Butler.

Spermatorrhœa. Its causes, symptoms, results and treatment. By Robert Bartholow, A. M. M. D. Fourth edition revised New York: William Wood & Co., Great Jones St., 1879. Buffalo: Peter Paul & Bro.

Hints in the Obstetric Procedure. By Wm. B. Atkinson, M. D., Physician to the department of Obstetrics and diseases of women, Howard Hospital, Philadelphia. Philadelphia: D. G. Brinton.

A Clinical treatise on diseases of the Liver. By Dr. Fried. Theod. Frerichs, Prof. of Clinical Medicine in the University of Berlin, etc., in three volumes. Vol. III translated by Charles Murchison M. D., F. R. C. P. New York: Wm. Wood & Co.

Lectures on Electricity in its relations to Medicine and Surgery. By A. D. Rockwell, A. M., M. D., Electro-Therapeutist to the N. Y. State Woman's Hospital. New York: Wm. Wood & Co.

Diphtheria, its nature and Treatment, varieties and local expressions. By Morell Mackenzie, M. D., London. Philadelphia: Lindsay & Blakiston.

Essentials of Chemistry, organic and inorganic, for the use of Students in need. By R. A. Withhaus, A. M., M. D. New York: Wm. Wood & Co. Buffalo: J. H. Matteson.

The Laws of Therapeutics, or the Science and Art of Medicine. By Joseph Kidd, M. D. Philadelphia: Lindsay & Blakiston, 1879. Buffalo: Theo. H. Butler.

Ophthalmic out-Patient Practice. By Charles Higgens, F. R. C. S. Second edition. Philadelphia: Lindsay & Blakiston. Buffalo: Theo. H. Butler.

Transactions of the American Gynecological Society; Vol. III for the year 1878. Boston: Houghton, Osgood & Company, 1879.

———:o:———

### Pamphlets Received.

Ninth Annual Report of the New York Opthalmic and Aural Institute.

Thirty-Third Annual Announcement of the Starling Medical College, Columbus, Ohio.

Urethrismus, or Chronic Spasmodic Stricture. By F. N. Otis, M. D.

Extracts from Twentieth Annual Report of Commissioners Lunacy of Scotland, for the year 1877, with an introduction. By H. B. Wilbur, M. D., Superintendent of N. Y. Asylum for Idiots.

Chloral Inebriety. Read before the Kings county Medical Society, April 15th, 1879. By J. B. Mattison, M. D., Brooklyn, N. Y.

College of Pharmacy of the City of New York. Prospectus Fiftieth Annual Course.

Thirtieth Annual Announcement of the Woman's Medical College of Pennsylvania. Philadelphia.

Albany Medical College, Medical Department of Union University, Annual Catalogue of 48th Session and Announcement for Session 1879–80.

Association of the Alumni of the Albany Medical College, Medical Department Union University. Proceedings of the Annual Meeting held Jan. 29th, 1879. Commencement Exercises and Dinner.

The relations of the Medical Profession to the State. Being the Anniversary Discourse delivered before the Medical Society of the State of New York. Feb. 5th, 1879. By D. B. St. John Roosa, M. D., President of the Society.

Annual Address. On the Relation of Neurasthenia to Diseases of the Womb. By William Goodell, M. D., Philadelphia, Pa.

On some points in Connection with the Treatment of Sterility. B A. Reeves Jackson, A. M., M. D., Chicago, Ill.

On the Traumatic Origin of Subfacial, Deep-seated, or cold abscess, commonly called Constitutional or Scrofulous Abscess. By Lewis A. Sayre, M. D.

Normal position and movements of the unimpregnated Uterus. By Ely Van De Walker, M. D.

Mechanical Restraint. By A. M. Shew, M. D., Superintendent Connecticut Hospital for the Insane.

Feigned Insanity. Report of case by E. N. Brush, M. D.

Mechanical Restraint in English Asylums. By W. Lander Lindsay, M. D. F. R. S. E.

Impotency in Women. By Ely Van De Walker, M. D.

9—7 4 cop/

### THE BUFFALO MEDICAL AND SURGICAL JOURNAL.

MEDICAL AND SURGICAL JOURNAL.

PUBLISHED MONTHLY,—Containing Original Articles, Reports of Medical Societies and Hospitals, Editorial, Reviews, Correspondence, Army News, etc., etc; including the usual variety of Medical Periodical Publications. Specimen copies sent on application to BUFFALO MEDICAL AND SURGICAL JOURNAL, J. F. MINER, M. D., 978 Main Street, BUFFALO, N. Y.

TERMS OF SUBSCRIPTION, $3 00 PER YEAR, IN ADVANCE,—All communications for publication should be sent before the fifteenth (15) of each month. or they will of necessity lie over until the next number. No change can be made in advertisements after the twenty-fifth.

Correspondence and original articles are solicited. Publication is no evidence that the views of the author are endorsed by the Journal.

